# The suitability and acceptability of a co-designed prototype psychoeducational activity book for autistic children aged five-eleven years

**DOI:** 10.1177/23969415241234648

**Published:** 2024-02-28

**Authors:** Lauren Powell, Gemma Wheeler, Chris Redford, Jonathan Stott

**Affiliations:** School of Education, 7315 University of Sheffield, Sheffield, UK; Children and Young People MedTech Co-operative, National Institute for Health and Care Research, London, UK; Chris Redford, Design and Illustration, Sheffield, UK; Child Orientated Mental Health Innovative Collaboration, 8748 University of York, York, UK; Tees, Esk and Wear Valleys NHS Foundation Trust, UK

**Keywords:** Autism spectrum disorders, school-age children, education, psychoeducation, participatory methods

## Abstract

**Background and aims:**

Evidence suggests that autistic children and young people (CAYP) can benefit from age-appropriate psychoeducation. Co-design is a methodology that iteratively involves end users and stakeholders in producing an intervention which may increase engagement and impact. Few age-appropriate co-designed psychoeducation resources for autistic CAYP exist. Therefore, a paper-based resource was co-designed for autistic CAYP who attend mainstream primary education. The resource aims to educate CAYP about their autism and provide strategies to support them to live well with their autism.

**Methods:**

This paper describes the evaluation of the prototype resource through online workshops with 12 families and input from four specialist clinicians. The suitability and acceptability of the resource was explored, and sketch notes were taken for respondent validity and engagement purposes.

**Results:**

A reflexive thematic analysis identified six themes and two subthemes: (1) content appropriateness (subtheme: strategies and unpredictability); (2) relating to content, (3) feelings and emotions, (4) terminology (subtheme: literal thinking); (5) positivity, and (6) communication aid. Suggested improvements were also identified. Results suggest the resource is suitable and acceptable.

**Conclusions:**

Future intervention development research may consider individual differences of autistic CAYP and the co-design of resources for other age groups of neurodiverse populations.

## Lay abstract

Autistic children can find it challenging to understand and live well with their autism. Challenges are often reported with regard to education, friendships, and communication. Research tells us that educating children about their autism in a way they understand, can be beneficial. However, information that is age-appropriate and accessible for autistic children is lacking.

Therefore, we worked with autistic children, their families/carers, and clinicians to design a paper-based activity book to help primary school-aged autistic children understand and live well with their autism. The activity book includes activities that aim to help them learn strategies to support autistic children and young people (CAYP) with challenges associated with their autism such as with friendships and communication. Eleven parents and 12 autistic children provided feedback on the activity book during online workshops. Four specialist clinicians reviewed electronic copies and provided verbal feedback.

Participants indicated the activity book was acceptable, suitable, and relatable to autistic children and provided improvement suggestions. They recognized the importance of understanding different words associated with autism such as “autistic” and “neurodiverse.” They also liked the positive tone of the activity book and used it to support important conversations between parents/carers and autistic children about their autism.

Following application of improvements suggested by our participants, the activity book has been shared with NHS Trusts, schools, and charities and is now being used with autistic children and families to learn about their autism and enable more of these important conversations between autistic children and their families.

## Introduction

Autism is a lifelong neurodevelopmental condition characterized by challenges in reciprocal social communication and social interaction, and restricted and repetitive behaviors (APA, 2013). And 1%–1.5% of people are diagnosed with autism across Europe (Bougeard et al., 2021). A UK cohort study found that increased awareness of autism has led to a 787% increase in autism diagnosis rates between 1998 and 2018 (Russell et al., 2022). Despite this, autism remains underdiagnosed in females (Hull et al., 2020). The lifetime cost of autism is around £1.5 million in the United Kingdom and is reported to be similar to costs of diabetes and attention deficit hyperactivity disorder (ADHD) and higher than costs for hypertension and stroke (Leigh & Du, 2015). Autism is a highly co-occurring condition (NICE, 2022), with significant overlap in the challenges experienced in ADHD (Ghirardi et al., 2018). Gender differences in presentation and diagnosis rates are also widely reported (Wood-Downie et al., 2021) with a ratio of 3:1 males:females obtaining a diagnosis (Bougeard et al., 2021).

The variable presentation of autism can include, social communication challenges, difficulties understanding other's emotions, interpreting information literally, making friends, and decreased interest in sharing with others (APA, 2013). Restricted and repetitive behaviors can include behavior and mental inflexibility, dislike of change in routine, overfocussing on individual interests at the exclusion of others, sensory hypersensitivity, and repetitive movements (APA, 2013). Mental flexibility refers to the ability to adjust thoughts and behavior in response to changing social situations (Hill, 2004). Additionally, autistic people can experience emotional regulation and executive functioning challenges which can contribute to poor academic attainment and challenges social interactions (Jahromi et al., 2013) and a lower quality of life than typically developing (TD) peers (Adams et al., 2019). Therefore, it is important to support autistic CAYP, especially as positive social interactions in childhood are associated with improved mental health, academic achievement, and peer acceptance (Hartup, 1989).

A UK online survey sought views and opinions of 3470 autistic people and their support networks around language to describe autism and found a preference for identity-first language (Kenny et al., 2016), which concurs with The National Autistic Society (2022) and NHS terminology recommendations (NHS, 2022). Therefore, identity first language will be used in this chapter.

A recent review recommends teaching autistic CAYP skills to help them live well with their autism and to compliment treatment plans, especially in relation to those who have experienced trauma (Peterson et al., 2019). Evidence favors a psychoeducational approach to compliment support for autistic CAYP (Powell et al., 2024). Definitions of psychoeducation are historically heterogeneous however this article defines psychoeducation as educating an individual about their condition, or somebody else's condition and learning skills (Powell et al., 2022) with the overall objective being to educate, support acceptance, and empower autistic CAYP (Beresford & Mukherjee, 2023). Benefits of psychoeducation can include improved wellbeing and quality of life (Warsi et al., 2004), which is particularly important for autistic CAYP as these factors are reported to be lower than in their TD peers (Adams et al., 2019). The UK clinical guidance states that best practice when sharing an autism diagnosis with CAYP should involve providing information for CAYP and their supporters and, if appropriate, should explain what autism is and how it could affect development and function (NICE, 2022). Unfortunately, there is a lack of postdiagnostic support for autistic CAYP, and families (Legg & Tickle, 2019) and psychoeducational support needs are not being met (Beresford & Mukherjee, 2023).

Varying attempts have been made to provide psychoeducation alongside support for autistic CAYP. Psychoeducation for autistic CAYP can include group and individual interventions, target different audiences such as educators and/or parents/carers, and can focus on acquiring knowledge and/or learning new skills, including social skills (Banach et al., 2010; Lucksted et al., 2012). Examples include clinic-based social groups (Olsson et al., 2017), support in school settings (de Bruin et al., 2013), support involving TD peers (Watkins et al., 2015), and parent social skills programs (Park et al., 2022).

Studies report effectiveness of psychoeducation support for autistic CAYP (Lee et al., 2009; Morsa et al., 2022) however most of this is aimed at supporters such as parents/carers rather than the CAYP. While there is value for educating support networks, it is important to educate the child as well as they often require support to develop life skills to enable them to live well with their autism (Wehmeyer & Shogren, 2008).

The challenges of existing psychoeducational support for autistic CAYP include mental flexibility challenges with generalizing skills learned to outside the context of the program (Olsson et al., 2017; Powell et al., 2022), as well as the expense of delivering resource intensive interventions which often involve CAYP visiting unfamiliar locations. This may create anxiety as a consequence of routine change, which is widely reported in relation to autistic people (Powell et al., 2024; Stark et al., 2021).

There is also little consistency around the content and delivery of existing psychoeducational support for autistic CAYP and little evidence or understanding of the educational needs of autistic CAYP (Morsa et al., 2022). Co-design methodologies could address this as they involve trained designers working with nondesigners throughout the creation process (Sanders & Stappers, 2008). In this instance, the nondesigners are the autistic CAYP, and their support networks (e.g., parents/carers, educators, and clinicians). This approach has been reported to lead to increased acceptability and impact (Greenhalgh et al., 2016) and has been successfully adopted to identify psychoeducational needs in CAYP with ADHD (Powell, Parker, et al., 2021). The authors used this approach to co-design a paper-based resource for primary aged autistic CAYP who attend a UK mainstream school. This study reports on the suitability and acceptability of this resource.

### The learning about autism activity book

Autistic CAYP (end users) and key stakeholders co-designed a “Learning About Autism Activity Book” for autistic CAYP aged between 5 to 11 years who attend a UK mainstream school. Stakeholders included families, education professionals, and specialist clinicians. The aim of the activity book is to help autistic CAYP understand and manage autism-related challenges. The detail of the previous co-design activities is beyond the scope of this article however a summary is provided below for context.

**Figure 1.**
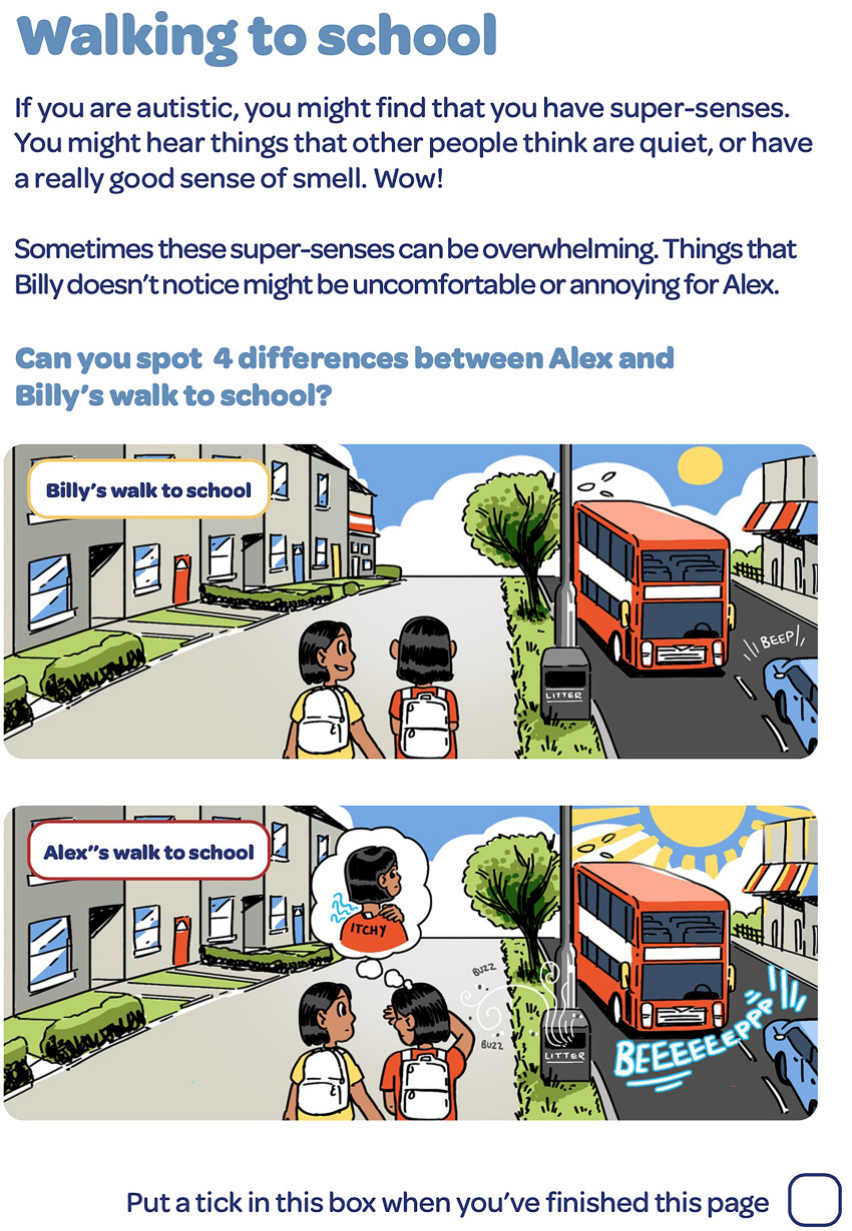
‘Spot the difference’ activity of Alex and Billy’s walk to school.

**Figure 2.**
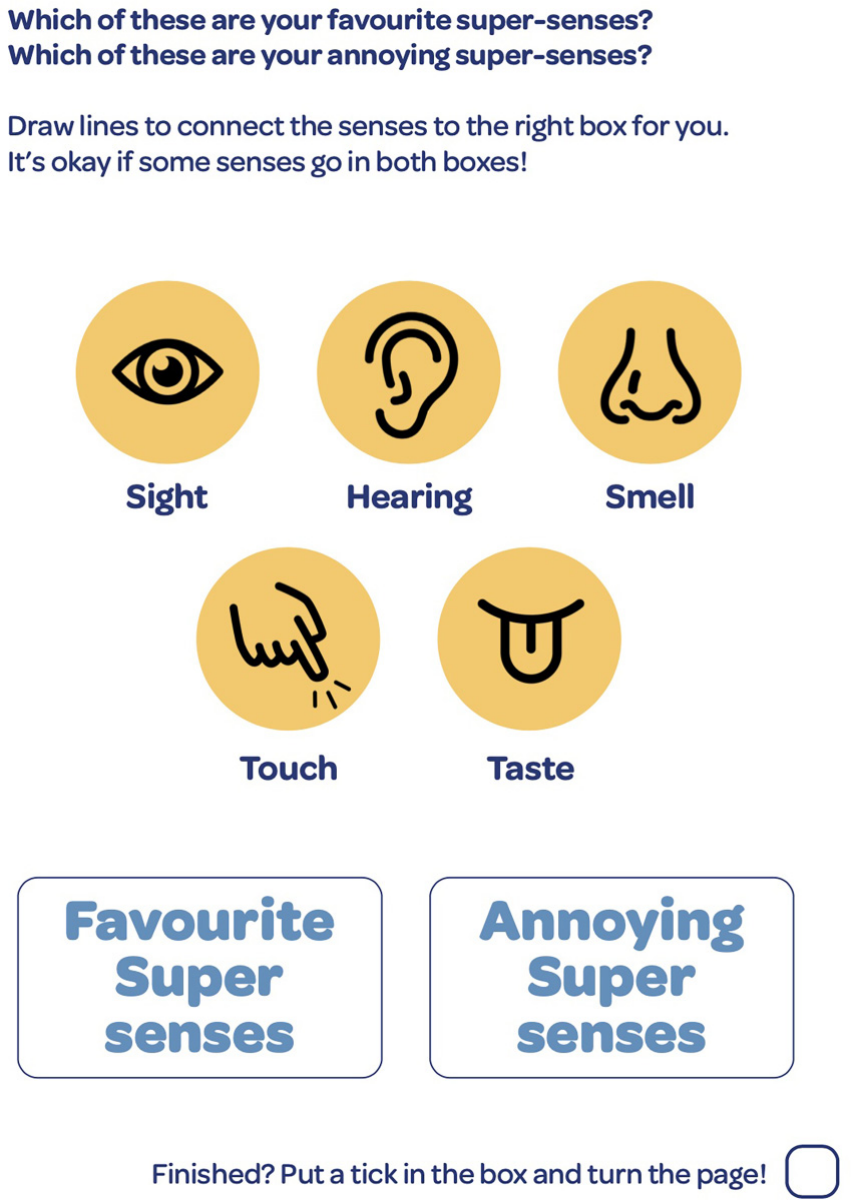
Activity to identify favorite senses (“super senses”) and senses that can be challenging for autistic CAYP. CAYP=children and young people.

**Figure 3.**
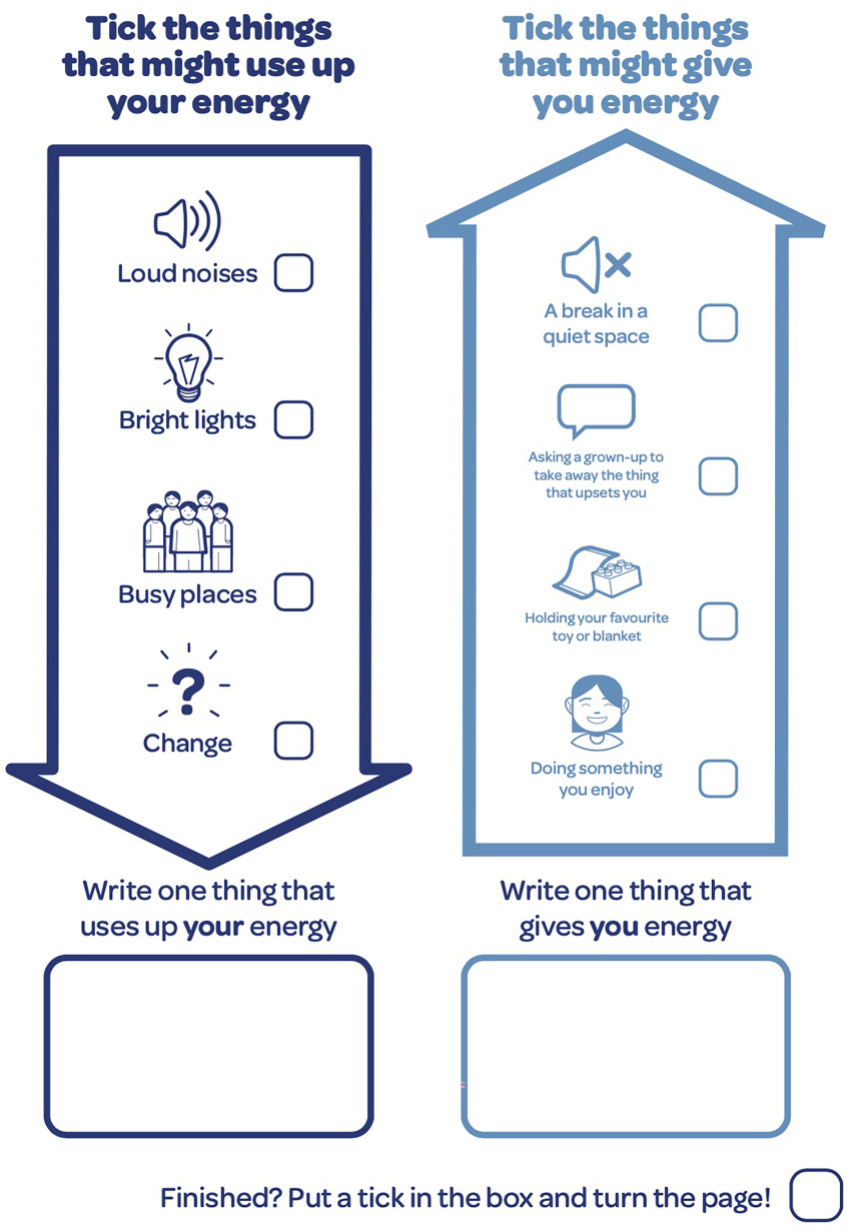
Activity where CAYP can acknowledge activities that may be draining and strategies to increase and/or preserve their energy levels. CAYP=children and young people.

Working alongside designers GW and CR throughout, resource development involved the use of postal activity packs, online workshops, and communications including in-person conversations, emails, and phone/video calls with end users and stakeholders. These activities confirmed the need for psychoeducation for autistic CAYP, the form the resource should take (paper-based activity book), and preferences of presentation of content. This led to the first activity book prototype. The activity book introduces twins, Alex who is autistic and Billy who is not. A day in the life of Alex and Billy is presented and demonstrates how Alex experiences the world differently to Billy because of their autism. Topics and activities explain what autism is, how to deal with change, routines, sensory challenges/benefits (“super senses”), what can drain and increase energy, recognizing emotions, taking turns and peer interaction (see Figures 1–3). The current version of the activity book is available on the ADHD Foundation website (Powell et al., 2021).

## Methodological approach

Authors of the present study believe it is important to be transparent about the positionality of the person analyzing and interpreting the data, especially as reflexive thematic analysis approaches view individual experiences and circumstances as a strength to data analysis and interpretation (Braun & Clarke, 2022). Author LP analyzed and interpreted the data. She is an adult female, who was diagnosed with ADHD and autism in adulthood. LP therefore allowed some of her experiences to resonate with participants meaning she could listen to them with empathy.

Therefore, a relativist ontological and subjectivist epistemological qualitative approach was adopted for this research whereby we assume views and opinions of our participants is based upon individual experiences and interpretations of these experiences (Hanly & Fitzpatrick Hanly, 2001; Jenkins, 2010). This complements a reflexive thematic analysis as this values individual experiences and how they influence interpretation of data, which does not have a single fixed meaning (Braun & Clarke, 2022). Here, open questions were asked during a series of workshops with autistic CAYP and their parents/carers. Additionally, feedback was obtained by professional stakeholders through a series of iterative phone calls and email communications.

Lastly, the approach to data analysis was deemed inductive as although authors sought views and opinions of the resource, they did not wish to force participants into commenting on predefined components of the resource, but more to provide in-depth information on the aspects of the resource they deemed important.

## Methods

Convenience sampling was adopted to recruit autistic CAYP (n = 11), their parents/carers (n = 11), and professional stakeholders (n = 5). Participants were recruited via a database that includes families who have expressed an interest in being involved in autism related research. Eligibility criteria for CAYP were aged 5–11 years, had a diagnosis of autism, and attended a UK mainstream primary school. Parents/carers had to be a parent of an autistic child attending a mainstream primary school in the United Kingdom. Stakeholders were defined as anybody who was involved with the support and or care of autistic CAYP in the United Kingdom.

### Procedures—children and families

Ethical approval was gained from author LP's institution (ref: 046063). Data collection was carried out remotely using Google Meet software and was adapted based on methods previously used with an ADHD population (Powell et al., 2021). This was deemed appropriate due to some of the overlapping needs in autistic CAYP and CAYP with ADHD (Ghirardi et al., 2018).

Participant information sheets, instructions on how to access Google Meet and two copies of the activity book were posted to participants prior to the Google Meet appointments. Participants were emailed a link to a Google form to provide informed consent, parental consent, and assent in advance of the appointment.

During the appointment, author LP went through the consent form and answered questions.

The Google Meet involved one family at a time, author LP delivering the workshop and note taking and author CR taking live drawings of topics discussed (“sketch notes”). The chat function was used to issue an emoji to recognize and celebrate when CAYP contributed to discussions. These emojis were added up at the end of the session and resulted in a treat (£10 Amazon gift voucher).

Author CR started with a “Zoom-in” game to practice the reward process described above. In the game CR shared his screen and zoomed in on an image of something the child was interested in (e.g., animals, superhero's). He then gradually zoomed out until the child guessed what the image was to gain an emoji. This was repeated for calls involving siblings to emphasize turn taking.

LP then asked open questions about their thoughts and opinions of the resource, while adjusting the pace and articulation of the questions based on the individual child and engagement levels. Question examples included “What is your opinion of the activity book?”; “Would you change anything about the activity book, if so, what would you change?”

Halfway through the call and at the end, CR shared the sketch notes for respondent validity purposes. Each Google Meet lasted up to 60 min with breaks. At the end of the call, LP, issued the gift voucher reward and emailed a copy of the sketch notes to the parents/carers.

### Professional stakeholders

Professional stakeholders were sent a study information sheet, consent form and prototype of the resource via email. They then signed the consent form and provided feedback on the resource to author LP via a series of phone calls, emails, and video calls.

### Data analysis

Demographic information was summarized (Table 1 and Table 2). Participant post codes were used to determine their Social Deprivation Index (SDI) to indicate current residential deprivation levels (Open Data Communities, 2020). This is indicated by a single figure, which quantifies levels of disadvantage across small areas, where higher levels of deprivation are indicated by higher numbers.

**Table 1.** Participant demographics of young people and parents.

Unique ID	Participant group	Gender young person	Child ethnicity	Social Deprivation Index	Child age (years)	Adult attending	When child diagnosed with Autism	Other relevant diagnosis
1	Parent only	Male	White British	24,514	10	Mother	2019	UAA, dyslexia
2	Parent and young person	Male	White British	16,261	9	Mother	2021	N/A
3	Young people 3 and 4: siblings One parent	Female	White British	24,463	6	Mother	2020	UAA
4		Male	White British	24,463	8	Mother	2018	N/A
5	Parent and young person	Male	White British	24,485	11	Father	2013	Sensory processing disorder, dyspraxia, UAA
6	Parent and young person	Male	Indian	28,549	8	Father	2018	ADHD
7	Parent and young person	Male	White British	27,532	8	Mother	2018	Tourette's, ADHD, demand avoidant profile, dyspraxia, anxiety
8	Parent and young person	Female	White British	2029	8	Mother	2019	ADHD, developmental delay
9	Parent and young person	Male	Black British	3695	9	Mother	2017	N/A
10	Parent and young person	Female	Arab	2476	10	Mother	2022	ADHD
11	Parent and young person	Female	White British	6101	11	Mother	2019	N/A
12	Parent and young person	Male	White British	22,786	11	Mother	2020	Dyspraxia

*Note*. Note that we have a sample that reflects residing in a variety of social economic areas. ADHD=attention deficit hyperactivity disorder; N/A=not applicable; UAA=under ADHD assessment.

**Table 2.** Participant demographics of stakeholders.

Unique ID	Profession	Gender
S1	Occupational Therapist	Female
S2	Pediatrician	Female
S3	Neurodisabiloity Nurse specialist	Female
S4	Psychiatry Registrar	Male

Participant identification key: YP=young autistic person; P=parent; S=stakeholder.

LP cross checked her written notes with CR's sketch notes. An inductive reflexive thematic analysis (Braun & Clarke, 2022) was adopted to seek patterns across data and involved six steps:

1. Data familiarization: This involved reading and reviewing the raw data.
2. Coding: This involved allocating short labels to the written and sketch note data. This was conducted by author LP in the first instance. In line with a reflexive thematic analysis, there was a single rather than multiple coders. This is because independent multiple coders adhering to a code book or coding frame are deemed to be underpinned by a realist/positivist approach, which is not consistent with the relativist/subjectivist approach to this study. This is because the realist/positivist approach would assume that there are fixed meanings within the data, and our approach acknowledges that interpretation of data can be subjective and are open to multiple interpretations (Braun & Clarke, 2022).
3. Searching for themes: This involved merging the labels (codes) from step two to begin to represent patterns across the dataset.
4. Reviewing themes: This involved discussions of identified patterns in the previous step with co-authors. In line with our reflexive thematic approach to data analysis, these discussions aimed to achieve a deepening of the authors; reflexive engagement of the data rather than checking accuracy of interpretations (Braun & Clarke, 2022).
5. Defining and naming themes involved agreeing with co-authors on what names should be allocated to themes that best represent their content and agreeing on descriptions of each theme.
6. Write up of results involved explaining these themes and using quotations from participants to evidence these themes. Quotations were chosen based on how clearly, they represented each theme. Authors also reviewed the quotations provided following report write up to ensure they represented the consensus of the data and came from a variety of participants.

Data were written notes and sketch notes alongside written responses from stakeholders and notes taken by LP from phone and video calls. Authors discussed and verified themes iteratively. Themes aimed to represent participants’ views and opinions of the suitability, acceptability, and improvements required of the resource.

### Community involvement

This research centers around the voices of autistic CAYP and their support networks to improve an already co-designed resource for autistic CAYP. All materials were reviewed by professional stakeholders to ensure information was correct and clinically appropriate before presenting it to CAYP and families. These reviews were undertaken by stakeholders from a variety of educational and clinical backgrounds, all of whom have experience of working with autistic CAYP.

## Results

**Figure 4.**
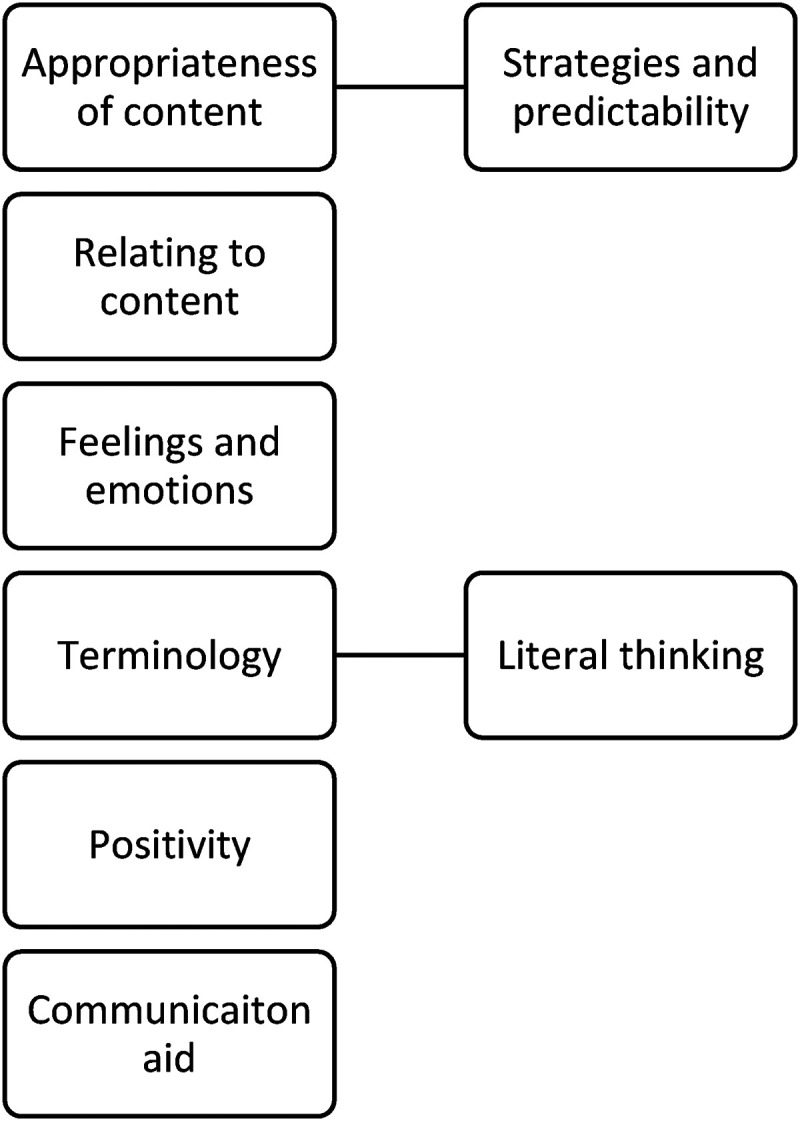
Identified themes and subthemes.

Following data familiarization, codes were allocated to the data. These codes included and were not limited to positive language, communication, literal interpretation, importance of visual information and relating to the content. The merging of these codes ultimately resulted in the identification of six themes and two subthemes (see Figure 4) that were deemed appropriate to capture the voices of the participants.

### Appropriateness of content

All participants agreed the content was appropriate. For example, a neurodisability nurse specialist and a YP with co-occurring dyspraxia stated they liked the presentation. The same YP, an autistic YP without diagnosed co-occurring conditions and a psychiatry registrar commented favorably on the colors. P1 and all stakeholders stated they liked the content and felt it was age and condition appropriate. One young person with Sensory Processing Disorder (SPD), dyspraxia and suspected ADHD stated that he thought the twins were “very well drawn” and liked that the graphics were like “a cartoon.” All parents commented favorably on the appropriateness of including activities so the CAYP could apply what they had learned to themselves. It was also noted that the resource was supportive for parents.

“*I like the colour and visual presentation… I think you address several of the challenges which are discussed in my clinic appointments” S3*

*“I think it's a very positive resource. [YP7] has responded well to it too. He's been looking at the rest of it. The activities have engaged him, and he seems to have enjoyed it.” P7*

Additionally, a neurodisability nurse specialist commented on liking the inclusion of sensory differences and all participants liked that there were activities for the CAYP to complete. An occupational therapist and psychiatry registrar commented on the “spot the difference” activity being a simple and appropriate way to convey information about sensory challenges.

*“…really glad you have included information about the sensory differences in autistic children and young people and we thought the “spot the difference” was a lovely simple way to help children understand it.” S1*

#### Strategies and predictability

Participants often noted the inclusion of potential strategies for autistic CAYP, which included ensuring their days are as predictable as possible to ease anxiety. All but one (YP9) of the CAYP stated that they related to the need for strategies to make their days more predictable. For example, all CAYP and parents apart from P9 and YP9 commented that the CAYP like to know what is happening each day in advance. P2, P5, and P12 thought that the option to complete a timetable for the day could be a useful strategy for autistic CAYP, particularly during weekends and holidays when there is less of an established routine. To that end, P11 noted that strategies provided in the resource to help calm autistic CAYP down are also useful.

‘*I think that's really good …because sometimes I think it's good for dad or mum to say what do you want to do or just tells me the complete plan for what we are going to do that day… stops me getting worried.’ YP5*

*“It's nice children can see other children use lists and gives parents idea of how to help” P7*

P12 noted that their child who also has a dyspraxia diagnosis often forgets to ask their friends questions and that the strategies provided for making friends could be helpful for autistic CAYP. P7 and YP11 acknowledged that providing autistic CAYP with strategies can be helpful. P7 also noted that the activity book can also act as a supportive resource for parents.

*“I like that it acknowledges doing something to give them energy as autistic people have to learn what works for them.” P7*

*“I think in some ways the activity book is already a teaching tool for parents too, giving them examples and ideas” P7*

### Relating to content

CAYP often commented favorably on content that they could relate to. For example, all but one (YP9/P9) of the CAYP and parents stated that they too dislike change. Three CAYP: two with dyspraxia, one of which also has a SPD diagnosis and an additional who did not have any co-occurring diagnoses, said they could relate to the challenges presented in the playground section of the resource and would like to try the suggested strategies for themselves with their friends. The young person without co-occurring diagnoses commented that the scenarios provided in the playground section are “actually true.”

Three CAYP, one of which was diagnosed with ADHD, one was under assessment for ADHD and one did not have any co-occurring conditions, wanted the front cover to be more personalized to them, perhaps by adding their name or including a space to draw something they like. Three CAYP: two with dyspraxia, one of which also has a SPD diagnosis and an additional who did not have any co-occurring diagnoses and P1 commented on how they could relate to the sensory challenges described in the resource and liked discussing this with their parents and completing the associated activities.

*“It is true that it can be hard to be in noisy places” YP11*

*“I am sure they (autistic children) would relate to this, it's absolutely fabulous !!!!!” S2*

### Feelings and emotions

**Figure 5.**
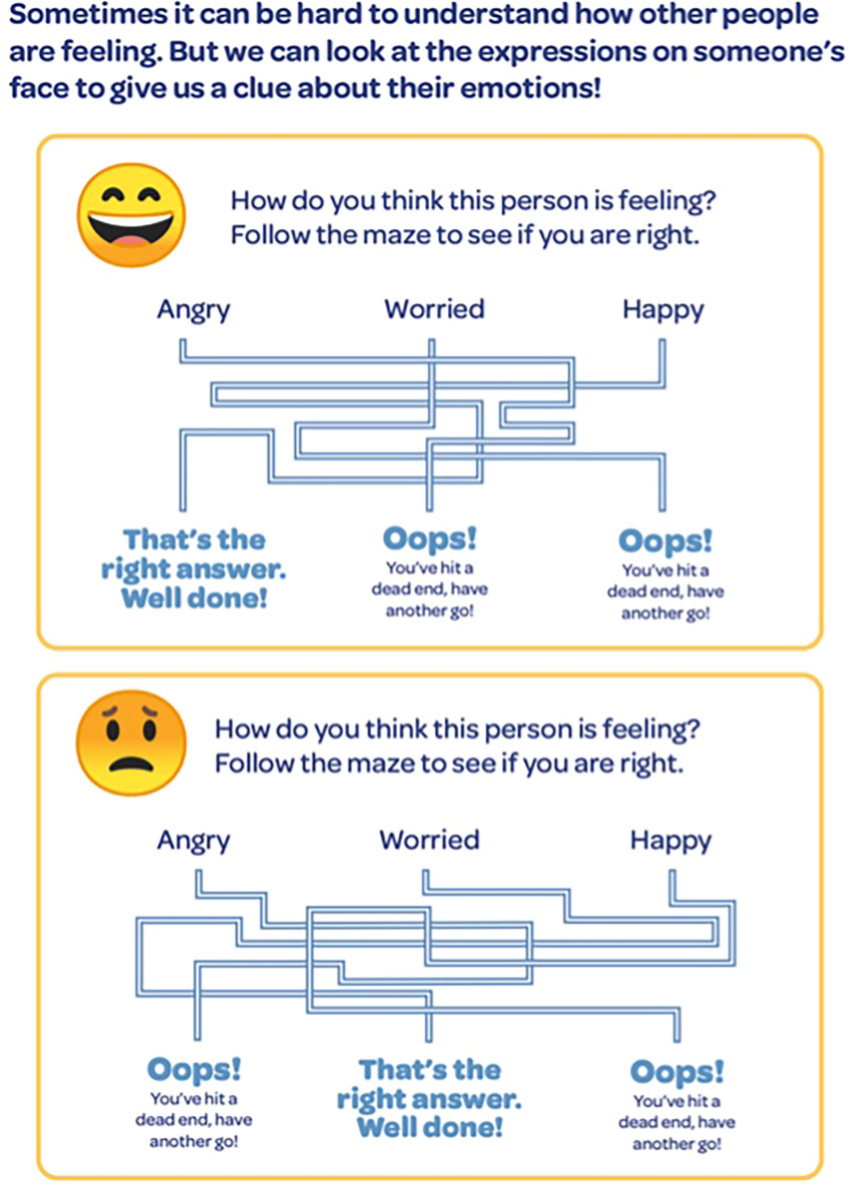
Emotion indication activity.

There was frequent discussion about feelings and emotions. In the resource there is a section with two emojis and an associated maze where CAYP are asked to indicate what the emotion the emoji is expressing (see Figure 5). P2 stated that their child finds it hard to talk about feelings and YP2 agreed. P2 and YP2 agreed that identifying happy and sad was easy but “identifying any emotion in between is a challenge” (P2). A YP with ADHD and another under ADHD assessment and with diagnoses of SPD and dyspraxia stated that the emojis should be bigger and YP11 and a psychiatry registrar commented on the usefulness of the emojis in demonstrating emotions. The psychiatry registrar also stated that this activity and the use of emojis could be argued to be useful to help the CAYP communicate with their peers.

*“…young people are communicating more and more using online technology and therefore often use emojis when communicating each other these days…” S4*

### Terminology

P1, 3, 5, 7, and 11, YP11 and a pediatrician commented on the usefulness of introducing terminology around autism, with P7 pointing out that language around autism is a particularly “contentious issues in the community.” They appreciated the explanation that *people use different words when they speak about autism* and that we use the term *autistic*; but other terms such as “person with autism” are okay and that they can use the words they prefer to use as there is no right or wrong answer.

*“I like how it says we can use different language around autism, and the reward idea is fantastic” YP5*

The psychiatry registrar felt that the introduction to terminology was age appropriate and easy for the CAYP to understand. In addition, a pediatrician felt that there could have been more terminology introduced here to get autistic CAYP used to the different words they may hear in relation to their autism.

“*I’d like a page that talks about the different words and ways others describe autism to help children get used to hearing different words e.g. ASD. Neurodiversity, sensory seeking etc” S2*

#### Literal thinking

This subtheme illustrates that some of the language appears to confuse some of the CAYP due to taking the language literally. For example, a bin with flies and words around the flies saying “buzz buzz” was intended to demonstrate a smelly bin. However, YP1 and 11 interpreted this as a noisy bin, due to the “buzz buzz” language.

When discussing what gives and takes away energy, YPs 3, 4, 6, 7, 8, and 10 confused the intended reference to mental energy with physical energy. It may also be useful to note here that these YP are all diagnosed with ADHD or under assessment for ADHD. One YP who was diagnosed with Tourette's sydrome, ADHD, dyspraxia, and anxiety acknowledged that where sitting in a quiet room was intended as a coping strategy to reinstate mental energy, they wondered why sitting in a room would give them more (physical) energy.

*“I think there is a confusion with YP7 between physical energy and what we mean.” P7*

### Positivity

P1, 5, and 7 commented on the overall positive language used within the resource and stated that language around autism isn’t always as positive as this and therefore they like this narrative. One YP who was diagnosed with SPD, dyspraxia, and was under assessment for ADHD, commented on liking the positive words used in the wordsearch and the words used to describe what makes autistic people amazing.

*“Best page in terms of making sure everyone knows they are amazing.” YP5*

P11 and the psychiatry registrar stated that they appreciated the acknowledgement that everyone is different and that this is positive. P11 liked the positive focus provided on the change of plan, which they said so many autistic CAYP struggle with. All the CAYP and parents commented that the space to write a reward for completing the activity book at the beginning and the certificate for completion at the end was a good idea and the CAYP liked the idea of cutting the certificate out to keep.

*“(The certificate) Reminder that you have achieved something.” YP11*

### Communication aid

P1, 2, 8, 10, and the psychiatry registrar commented that the resource acts as a communication aid between the parent and the young person, enabling conversations about their autism that may not be possible without an evidence based and trustworthy resource.

*“It's valuable doing the activities with an adult to enable conversations (about their child's autism).” P2*

P12 also commented that it may help them to make friends at school in the playground by giving them ideas around how they can speak to their friends, that is, ask friends questions and do not only talk about yourself, and to ask if they are okay if you are not sure. P9 stated that YP9 now understands his autism thanks to this project and the resource.

### Implementation

Parents and clinicians identified and agreed on how the resource could be used in practice. For example, the consensus was that the resource could be used by autistic CAYP who were already diagnosed and those receiving a new diagnosis. For these populations to access the resource, parents, and clinicians agreed that it should be freely available on charity and NHS websites and where practicable, paper copies could be made available via NHS services, schools, educational psychologists, and assistants in social care settings and charities and organizations who work with families with autistic CAYP. It was also suggested that where practical, schools could provide the resource to individual families, use in class or with special educational needs coordinators on a 1–1 basis.

### Suggested improvements

Participants made some specific suggestions to improve the resource prototype, which have subsequently been applied to the final version. For example, they wanted some small alterations to personalize the resource to the CAYP such as a space to write the CAYPs name on the front cover, additional terminology to be added such as “neurodiverse” and “ASD.” Some interactive components such as asking the child “What questions could you ask your friends when you talk to them?” on the comic strip section. There were places that could have been clearer to the CAYP, so changes were requested such as emphasizing an image of a bin was smelly rather than noisy as it had flies buzzing around it originally. Emojis were also requested to be larger and therefore clearer. CAYP also requested that the size of a game a character is reduced as originally, they were the same size as the game they were playing, which was noted as not realistic.

### Live sketch note observations

**Figure 6.**
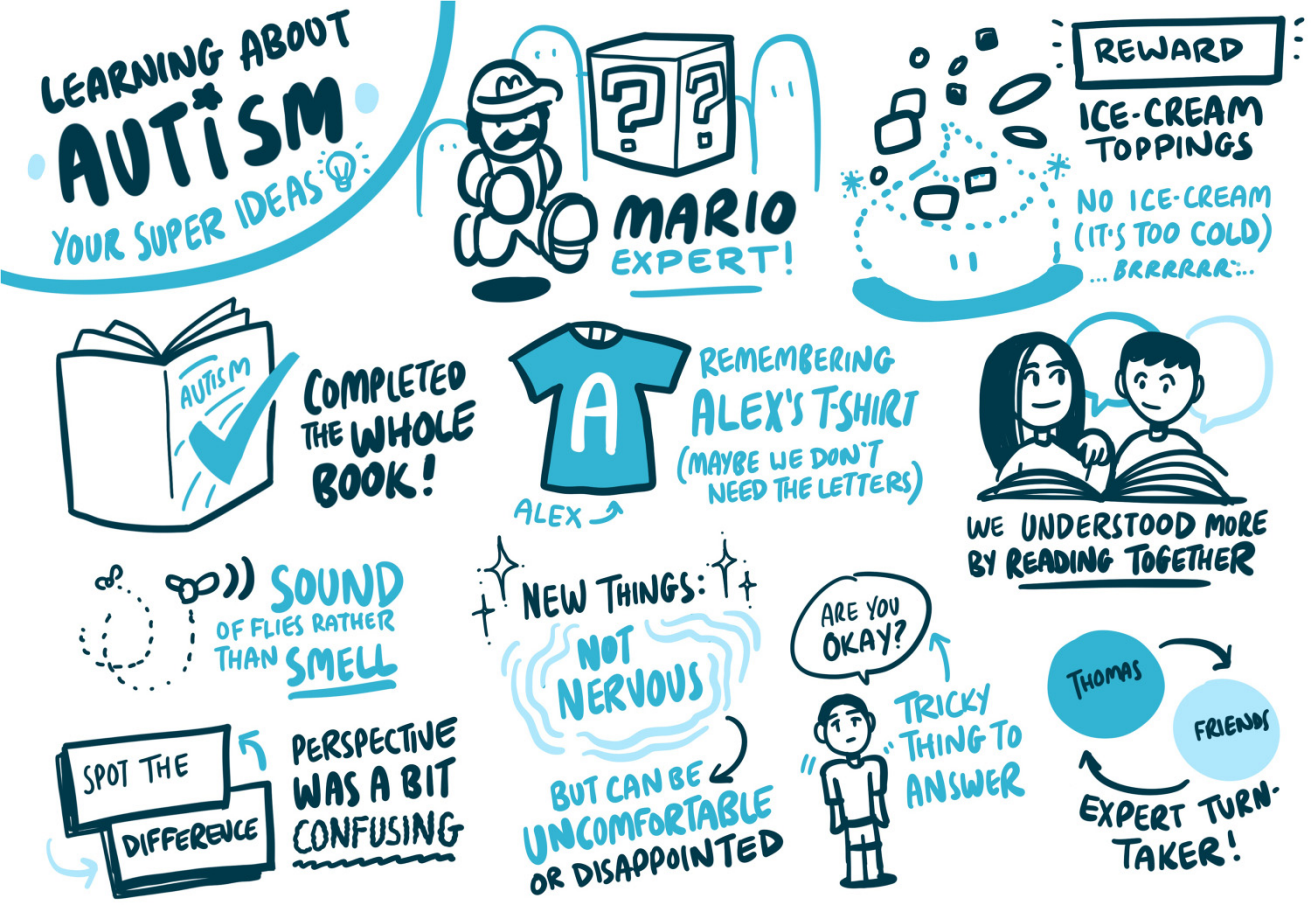
Sketch notes from P2.

**Figure 7.**
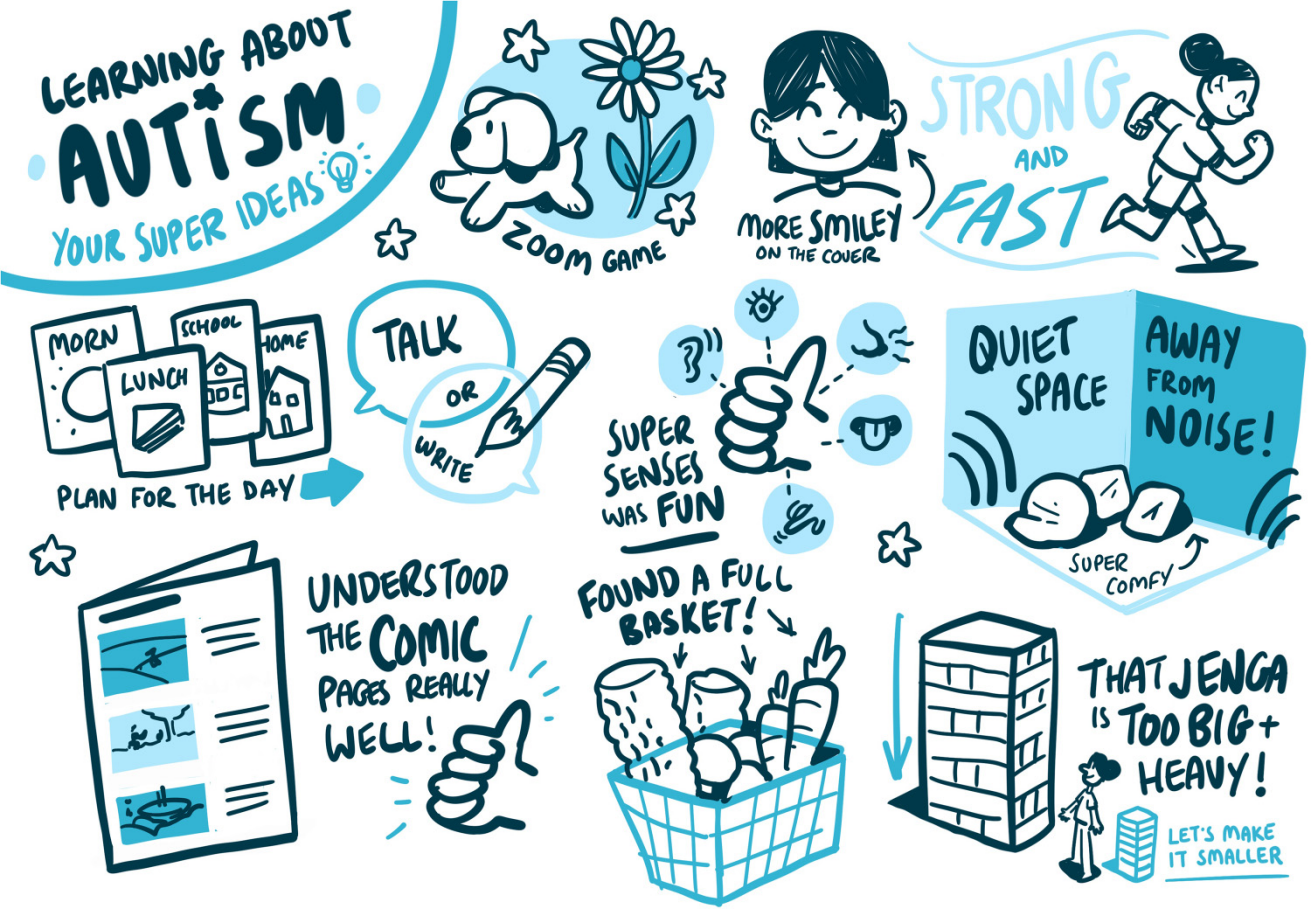
Sketch notes from P8.

YP1–11 received sketch notes. YP12 and P12 chose a date the graphic designer was unavailable, a choice they were happy with. Sketch notes were used to record what participants said, to ensure respondent validity and maintain engagement during the video calls. All CAYP and parents who received sketch notes spoke favorably of them. Examples of sketch notes can be found in Figures 6 and 7.

## Discussion

This study aimed to explore the suitability and acceptability of a new autism psychoeducation resource for primary school-aged autistic people who attended mainstream. Our study also aimed to document any suggested changed for improving the resource. Six themes and two subthemes emerged from the data and suggested that the resource is suitable for the intended population. Participants provided a list of suggestions to improve the prototype.

Previous research indicates that age appropriate, interactive, and visually appealing content are vital for the suitability of psychoeducational resources for CAYP with ADHD (Powell et al., 2021). This was echoed by our participants who deemed the content of the resource as suitable for the intended population and were therefore willing to share with their children/patients. Participants in this study also indicated a preference for being able to personalize the content, something previous research with CAYP with ADHD has also reported (Powell et al., 2021).

All participant groups in this study appreciated the positive identity-first language used in the resource to describe and refer to autism. This linguistic preference is also indicated in research (Kenny et al., 2016) and endorsed by the National Autistic Society (2022) and the NHS (2022). However, there were places whereby language caused some confusion due to the autistic CAYP's unintended literal interpretation of some of the content, something that is often reported in the autistic community (Hobson, 2012). This evidences that it is vital to include autistic CAYP in this process to ensure the acceptability and suitability of the final resource.

In terms of the adoption of positive language in the resource, this is important as it is widely reported that autistic people and their families often experience stigma toward their autism (Cage et al., 2019). Changing the often negative and stigmatized narrative is important to improve inclusivity (Turnock et al., 2022), employment opportunities for autistic people (Djela, 2021), and empower autistic people and their families (Cleary et al., 2023).

Existing research acknowledges the paucity of available reliable information about their child's autism (Nussey et al., 2013). Therefore, it is perhaps not surprising that parents/carers in this study reported that the positive language and reliable content gave them the confidence to have important and empowering conversations with their child about their child's autism, often for the first time. Similarly, this was echoed by the CAYP, who appreciated the strategies provided in the resource to support conversations about their autism with their peers, a common challenge for autistic CAYP (Finke, 2016) and something that was identified by autistic CAYP in our public engagement work in earlier stages of development. This finding has been mirrored previously in CAYP with ADHD and their parents/carers (Powell et al., 2021).

Further to the language use in the resource, all participant groups in this study appreciated the communication strategies provided such as encouraging autistic CAYP to ask their peers questions about them rather than only talking about themselves. Wider literature supports the need for such strategies as it is widely reported that autistic CAYP find it difficult to make friends, struggle with but still wish to engage in social interactions (Jahromi et al., 2013; Levi et al., 2023). This is crucial because positive social interactions are reported to be associated with improved mental health, academic achievement, peer acceptance, and improved scholastic achievement (Hartup, 1989; Lord et al., 2018).

In addition to recognizing the reliability of the content of the resource, participants noted the appreciation of relating to the content. For example, the resource explains how autistic CAYP often prefer predictability and routine and acknowledges that change from routine can be anxiety provoking (Stark et al., 2021). It also recognizes that change is not always negative, which was particularly welcomed by the parents/carers in this study.

All participant groups commented favorably on how the resource explains that sensory sensitivities in autism can provide challenges, which is also supported by existing evidence and clinical guidance (Ben-Sasson et al., 2009; Little et al., 2018; NICE, 2022; Tomchek et al., 2014). Our participants reported they particularly liked the emphasis on how these sensitivities can be challenging as well as beneficial, for example, some sensory experiences can be overwhelming, and others can be comforting and pleasurable.

CAYP often commented that they found feelings and emotions difficult to talk about. Reasons were mostly identified by parents and included lack of understanding emotions other than happy and sad and difficulty articulating the emotions they are feeling. It is widely reported that autistic people can experience difficulty identifying others’ emotions (APA, 2013). This can be explained by the Theory of Mind, which refers to a cognitive skill whereby individuals attribute mental states to themselves or other people, which is fundamental for social functioning and understanding others’ behaviors, something that is reported to often be a challenge for autistic people (Baron-Cohen et al., 2001).

To address this the resource adopted the use of emojis. One clinician noted that this could help CAYP think about identifying emotions is useful due to the increased reliance on technology for CAYP to communicate with each other. Research across different cultures has noted the increased use of emojis and their potential usefulness for communicating especially with young children, including on a variety of topics from food-related emotions to attitudes and maths (Gallo et al., 2017; Huang et al., 2022; Massey, 2022). Emojis have been argued to be a useful tool for supporting young children to have a voice in research (Fane et al., 2018). Further, recent research has found that autistic CAYP are able to identify emojis however if they also have alexithymia, the lack of ability to identify and describe emotions in oneself or others (Bird & Cook, 2013), they were less accurate at identifying emoji emotions than if they also had attachment and anxiety challenges (Taylor et al., 2022). Therefore, individual differences within the autistic population could influence their ability to interpret emojis successfully.

Lastly, this resource is ultimately aiming to aid the understanding of autism in autistic CAYP and provide strategies to help them live well with their autism. This will often require a level of behavior change. Results from this study indicate that autistic CAYP are engaged by the resource for the reasons discussed above. Therefore, it could be concluded that the resource provides autistic CAYP with the capability and motivation to implement some of the suggested strategies, both of which have been identified as integral for any behavior change (Michie et al., 2011).

### Study strengths and limitations

The sample size is limited, and results should be generalized with caution. This low number of participants did however allow detailed data collection, necessary in exploring the research question. Subject response bias is possible though opinions provided were balanced and useful to improve the resource. Education professionals were not included in the present research, arguably narrowing the perspectives. However, they were heavily involved in previous co-design activities referred to in the Learning About Autism Activity Book section above. Therefore, their views and opinions have been considered.

The live sketch notes allowed the researchers to engage the YP by sharing the notes as they progressed at intervals during the workshops, and this also enabled the accuracy of respondent validity to be verified with the young people. This was deemed a more accessible way to confirm respondent validity for this population compared to sending written transcripts.

The authors hope the resource will potentially help improve existing knowledge, which could lead to further important conversations between parents/carers and their autistic CAYP, empowering autistic CAYP and providing them with strategies to manage autism related challenges. The authors acknowledge that the resource will not be suitable for all autistic CAYP who attend a mainstream school. Resource distributors will need to use their expert judgment on an individual basis. This has been achieved previously for a resource for CAYP with ADHD (Powell et al., 2021).

### Future research

Future research that develops psychoeducation for neurodiverse CAYP should recognize and acknowledge their content as psychoeducation. There is a plethora of research that includes development of psychoeducation for CAYP with neurodisabilities but omits the term psychoeducation. Reasons for this may include the variety of professional backgrounds and expertise of teams conducting such research such as clinicians, education professionals, and psychologists and the variety of terminology that is accepted and used in these disciplines. Further, psychoeducation is not something that is often delivered as a stand-alone intervention or approach. For example, there are often psychosocial interventions that include psychoeducational components but do not describe it using this terminology (Beaumont et al., 2021; Begeer et al., 2015; Kent et al., 2020; Koning et al., 2013; Lopata et al., 2018, 2021; Nowell et al., 2019; Shih et al., 2019; Soorya et al., 2015; Thomeer et al., 2019). Despite the historically heterogeneous tendency to define psychoeducation, many definitions often agree that the overall aim is to educate somebody about a condition and how to live well with it (Beresford & Mukherjee, 2023). Therefore, the defining features of psychoeducation could be argued to be learning what a psychological (rather than physical) condition is and provision of tools to live well with that condition. This rather broad nature of psychoeducation could also explain the lack of terminology consistency in the literature, thus creating the difficulty in identification of such work which could limit important learning that may be integral to future research development.

Future studies should also acknowledge the information needs of their intended audiences as this study has shown how important it is to gain continued and repeated input from both the stakeholders and the end users. The live sketch notes were a valuable tool for this work, something future research with autistic CAYP may benefit from. This approach did involve identification and payment of a graphic designer, therefore if sketch notes planned to be incorporated into research, consideration must be made when planning the work and the budget.

Further, work in this area should consider and be transparent about the co-occurring diagnoses of their participants. Participants in the present study had a range of co-occurring diagnoses (see Table 1), which is a realistic representation of the autistic population. However, it is possible that different diagnoses could dictate the varying needs of the young person. Future research could explore the possibility of resources for CAYP with combined autism and ADHD diagnoses however great care should be taken here due to the different needs of this population. That is, although there is often considerable overlap in how autism and ADHD present, it is well documented that the underlying factors influencing observable symptoms in autism and ADHD can differ. For example, research indicates that there are differences in both general and social reward processing, social cognition, and brain connectivity (Taurines et al., 2012).

### Implications and recommendations

Following improvements identified in this study, the resource has been accepted by gatekeepers. This is likely because it was co-designed and included end users and stakeholders at every development stage. As a result of this acceptance, the resource has been distributed to NHS Trusts, schools, and charities across the United Kingdom.

The UK clinical guidance states that best practice when sharing an autism diagnosis with CAYP and their supporters should involve sharing information with the CAYP, if appropriate, that explains what autism is and how it may affect development and function (NICE, 2022). This is also supported by wider evidence such as previous work that recommends teaching autistic CAYP skills to help them manage autism-related challenges can complement treatment plans (Peterson et al., 2019). Our participants supported this and stated they believed the resource could be useful for CAYP when diagnosed with autism and with CAYP with an existing autism diagnosis.

The importance of this is firmly supported by other work that emphasizes how important it is to educate CAYP about their condition because as they grow up, autistic CAYP often require support to develop life skills to enable them to live well with their autism (Wehmeyer & Shogren, 2008).

The resource could also be helpful to provide a baseline understanding of autism and therefore reduce clinic time by ensuring questions asked are more targeted. It is however not intended to replace clinic time but to compliment it. This is something that was noted by clinicians in our early public involvement work.

## Conclusions

Participants indicated the resource is acceptable and suitable for autistic CAYP attending mainstream primary education. Reasons for this included and were not limited to the reliable content, identity first and positive use of language, strategies provided to support communication between autistic CAYP, their families and peers and the content that was perceived to be relatable for autistic CAYP. The consensus was that the resource could be used with CAYP with a new and an existing autism diagnosis via health and social care services and education settings. This study also highlighted the importance in utilizing methodologies that require continued involvement of stakeholders and end users in resource development. Next steps could involve the adaptation of the resource for autistic CAYP with learning disabilities who perhaps do not attend mainstream education and to continue to develop psychoeducational resources for CAYP with neurodisabilities in partnership with the intended audiences and their supporters.
